# The median effective dose of ciprofol combined with a low-dose sufentanil for gastroscopy in obese or nonobese patients: a dose-finding study using Dixon’s up-and-down method

**DOI:** 10.3389/fphar.2025.1521715

**Published:** 2025-02-18

**Authors:** Jie Zhao, Yixiao Zhang, Guowei Su, Shaoyi Wang, Xiaolin Zhang, Guoxiang Wang, Gang Chen

**Affiliations:** ^1^ Department of Anesthesiology, Sir Run Run Shaw Hospital, Zhejiang University School of Medicine, Hangzhou, China; ^2^ Department of Anesthesiology, Hangzhou Red Cross Hospital, Hangzhou, China

**Keywords:** ciprofol, patients with obesity, painless gastroscopy, sufentanil, dosage exploration

## Abstract

**Objectives:**

Understanding the different pharmacodynamic responses to narcotics in patients with or without obesity is particularly important for the safety of gastroscopy sedation. This study aimed to determine the median effective dose (ED50) of ciprofol combined with low-dose sufentanil to inhibit the response to gastroscope insertion in obese or nonobese patients.

**Methods:**

A total of 27 obese patients (BMI 30–40 kg/m2) and 25 nonobese patients (BMI 18–25 kg/m2), aged between 18 and 65 years, with ASA physical status of 1–2, were included in this study. All patients underwent painless gastroscopy and received intravenous sufentanil at a dose of 0.1 μg/kg, followed by ciprofol administration. The initial dose of ciprofol for the first patient in both groups was 0.4 mg/kg, the subsequent dose was determined by the response of the previous patient to gastroscope insertion (cough, choking, body movement, etc.) using Dixon’s up-and-down method. The dose was increased or decreased by 0.05 mg/kg depending on the observed responses. Data collection continued until 7 crossover points were obtained. Probit regression and bootstrapping methods were employed to calculate the median effective dose (ED50) and 95% confidence intervals (CIs). The ED50 values were then compared between the obese and nonobese patient groups.

**Results:**

The ED50 of ciprofol combined with sufentanil inhibiting response to gastroscope insertion in patients with obesity was 0.186 mg/kg with 95% CI of 0.153∼0.209 mg/kg, was significantly lower than patients with nonobese was 0.237 mg/kg with 95% CI of 0.206∼0.253 mg/kg (p < 0.05).

**Conclusion:**

The ED50 values of ciprofol combined with sufentanil inhibiting response to gastroscope insertion in patients with obesity was lower than in patients with normal weight.

**Trial registration:**

https://www.chictr.org.cn/bin/project/edit?pid=202873, identifier ChiCTR2300074216.

## 1 Introduction

With growing public concern regarding health issues, painless gastroscopy has become increasingly important for obese patients as part of their routine health examinations (Zheng et al., 2023; Yan H. et al., 2023). However, these patients often experience respiratory depression, airway obstruction, and subsequent hypoxemia during painless gastrointestinal endoscopy due to their unique anatomical and physiological changes, including altered metabolic and pathophysiological states (Yan L. et al., 2023; Chen et al., 2022). Furthermore, the increased volume of distribution (Vd) observed in obese patients for drugs with high lipid affinity may lead to dosing challenges (Wu et al., 2021). Improper dosage of anesthesia medications in obese patients increases the risk of adverse events and suboptimal efficacy. Given these considerations, it is important to carefully select appropriate anesthetic drugs and accurately determine the optimal drug dosage for people of different weights.

Previous studies have utilized propofol in combination with sufentanil as a sedation strategy for painless gastroscopy. However, propofol carries a higher risk for obese patients due to their increased susceptibility to airway collapse and cardiopulmonary complications. This has prompted the exploration of alternative sedative agents that may be better suited for obese patients (Xiao et al., 2022; Young et al., 2002; Wani et al., 2011). Recently, Ciprofol (also known as HSK3486) emerged as a potential candidate that may be more suitable for sedation in obese patients. It has been reported to possess the potency of propofol but at a reduced dosage, making it an attractive option for anesthesia management in this patient population (Qin et al., 2017; Zhang et al., 2017; Wei et al., 2017). We therefore hypothesized utilizing ciprofol combined with sufentanil, could mitigate the airway and cardiopulmonary-related risks in obese patients undergoing painless gastroscopy. Ciprofol has been approved for use in sedation and anesthesia during non-tracheal intubation or procedures, induction and maintenance of general anesthesia, and sedation during intensive care procedures (Lu et al., 2023; Liu et al., 2021; Liu et al., 2022; Teng et al., 2021; Li et al., 2022; Zhong et al., 2023). However, there is a lack of sufficient studies investigating the pharmacodynamic effects of ciprofol in obese patients, and limited data are available regarding the weight-based dose of ciprofol in this specific population.

Understanding the median effective dose (ED50) of ciprofol in both obese and non-obese patients is crucial for optimizing sedation protocols during painless gastroscopy. This study aimed to determine the ED50 (median effective dose) of the inhibitory response to ciprofol in combination with low-dose sufentanil during gastroscopy insertion in patients with obesity and nonobese controls for sedation.

## 2 Material and methods

### 2.1 Clinical registration and ethics approval

This study was registered on the Chinese Clinical Trial Registry, a primary registry of the WHO international clinical trial registry platform (ChiCTR2300074216, Registration Date: 1 August 2023). The research study was approved by the institutional review board of Hangzhou Red Cross Hospital (NO.2023-095) and conducted in accordance with the ethical guidelines of the 1975 revision of the Declaration of Helsinki. All patients in our study provided written informed consent.

### 2.2 Criteria for inclusion and exclusion

All patients undergoing elective gastroscopy from August to December 2023 were enrolled in the study. Patients were included if they met the following criteria: age between 18 and 65 years, body mass index (BMI) either between 18 and 25 kg/m2 or 30 and 40 kg/m2, and ASA physical status I or II.

Exclusion criteria included American Society of Anesthesiologists (ASA) physical status 3 or higher; contraindication for sedation or anesthesia or a history of sedation/anesthesia adverse events (AEs); patients with severe hypertension, diabetes mellitus, hepatic and renal dysfunction, abnormal thyroid function or other diseases that may affect pharmacokinetics; Patients with difficult airways, sleep apnea syndrome, and subjects exhibiting significant respiratory and cardiovascular dysfunction; lactating or pregnant women; and allergy to any of the study medications.

### 2.3 Anesthesia protocol and endoscopic procedure

Before the painless gastroscopy, all patients were fasted for at least 8 h and no water for 2 h. After admission, blood pressure (BP), heart rate (HR), and pulse oxygen saturation (SpO2) were monitored continuously. Intravenous access was established and put on oxygen at 4 L/min through a nasal straw.

The same anesthesiologist performed all Anesthesia operations. Patients were administered intravenous 0.1 ug/kg sufentanil 30 s and ciprofol with 1 mL/s for sedation. The sequential dose was assigned according to Dixon’s up-and-down method, with an initial dose of 0.4 mg/kg for the first patient in both groups with and without obesity. Patients who could not tolerate gastroscopy within 5 min of the injection of ciprofol, as evidenced by coughing, choking, or any physical movement during the placement of the gastroscope, were considered “responsive” and a single dose of 0.1 mg of ciprofol was administered intravenously and repeated to complete the gastroscopy. Accordingly, the dosage of ciprofol for the next patient was increased by a step size of 0.05 mg/kg, and if the gastroscopic examination was successfully completed, the dose for the next patient to be examined was decreased by 0.05 mg/kg. The corresponding ciprofol dose at the midpoint of responsive and non-responsive was defined as the effective dose of ciprofol for 1 crossover, and at seven crossover points were obtained before the conclusion of the study. The Modified Observer’s Assessment of Alertness/Sedation Scale (MOAA/S) was used for measuring the depth of sedation. Gastroscopy started when MOAA/S ≤ 1 points and the MOAA/S was assessed every minute during gastroscopy by an experienced anesthesiologist, who was blinded to the study group.

If SpO2 decreases, airway intervention should be initiated based on the severity of the decline. Specific measures include: increasing the flow of inhaled oxygen to 8 L/min when SpO2 falls below 95%; if SpO2 drops below 90%, the anesthesiologist would lift the jaw to relieve upper airway obstruction. For SpO2 levels below 85%, a nasopharyngeal airway should be inserted. If oxygen saturation does not improve despite these measures, the decision to remove the gastroscope and proceed with mask ventilation or even tracheal intubation should be made depending on the degree of hypoxia. Ephedrine injection of 6 mg was given intravenously when the blood pressure decreased by more than 30% compared with the baseline value, or the mean arterial pressure was less than 60 mmHg. Atropine (0.5 mg) was administered if patients had bradycardia (HR < 50 bpm).

### 2.4 Outcome assessments

The primary outcome of the study was to determine the median effective dose (ED50) of ciprofol in combination with low-dose sufentanil for obese or nonobese patients using Dixon’s up-and-down method.

Secondary outcomes were the occurrence of adverse events (hypoxemia, hypotension, bradycardia, choke, and apnoea) and satisfaction scores in the obese and nonobese groups; Hypoxemia is defined as SpO2 <90% for >10 s. Changes in systolic blood pressure (SBP), diastolic blood pressure (DBP), oxygen saturation (SpO2), and heart rate (HR) before induction of anesthesia (T0), 1 min after induction (T1), at the time of gastroscope insertion (T2), at the time of 3 min of examination (T3), at the time of withdrawal of gastroscope (T4), and at the time of awakening (T5) in both groups.

### 2.5 Statistical analysis

The required sample size was calculated using Dixon’s up-and-down method (Dixon, 1991). This method requires at least six crossover points (non-responsive to responsive) for statistical analysis. Probit regression model analysis was used to derive the drug dose required for the sedative effects of ED50 of ciprofol based on modeling the response to different dose levels based on binary outcomes. 95% confidence intervals (CIs) for ED50 was derived using the bootstrap method (Bootstrap). The comparison of ED50 values is determined by the overlapping region of the 95% confidence intervals to assess whether there is a significant difference.

Continuous data were tested for normality using the Kolmogorov–Smirnov method and presented as mean (standard deviation [sd]) or median (inter-quartile range) as appropriate. Normally distributed data were analyzed using an independent-sample t-test. Non-normally distributed data were analyzed using the Mann-Whitney U test.

Categorical variables were expressed as frequencies (%) and compared with Fischer’s exact test. All analyses were performed using R version 3.4.1 (R Foundation for Statistical Computing) and GraphPad Prism version 5.0 (GraphPad Software Inc., San Diego, CA, United States). P < 0.05 was considered statistically significant.

## 3 Results

**FIGURE 1:**
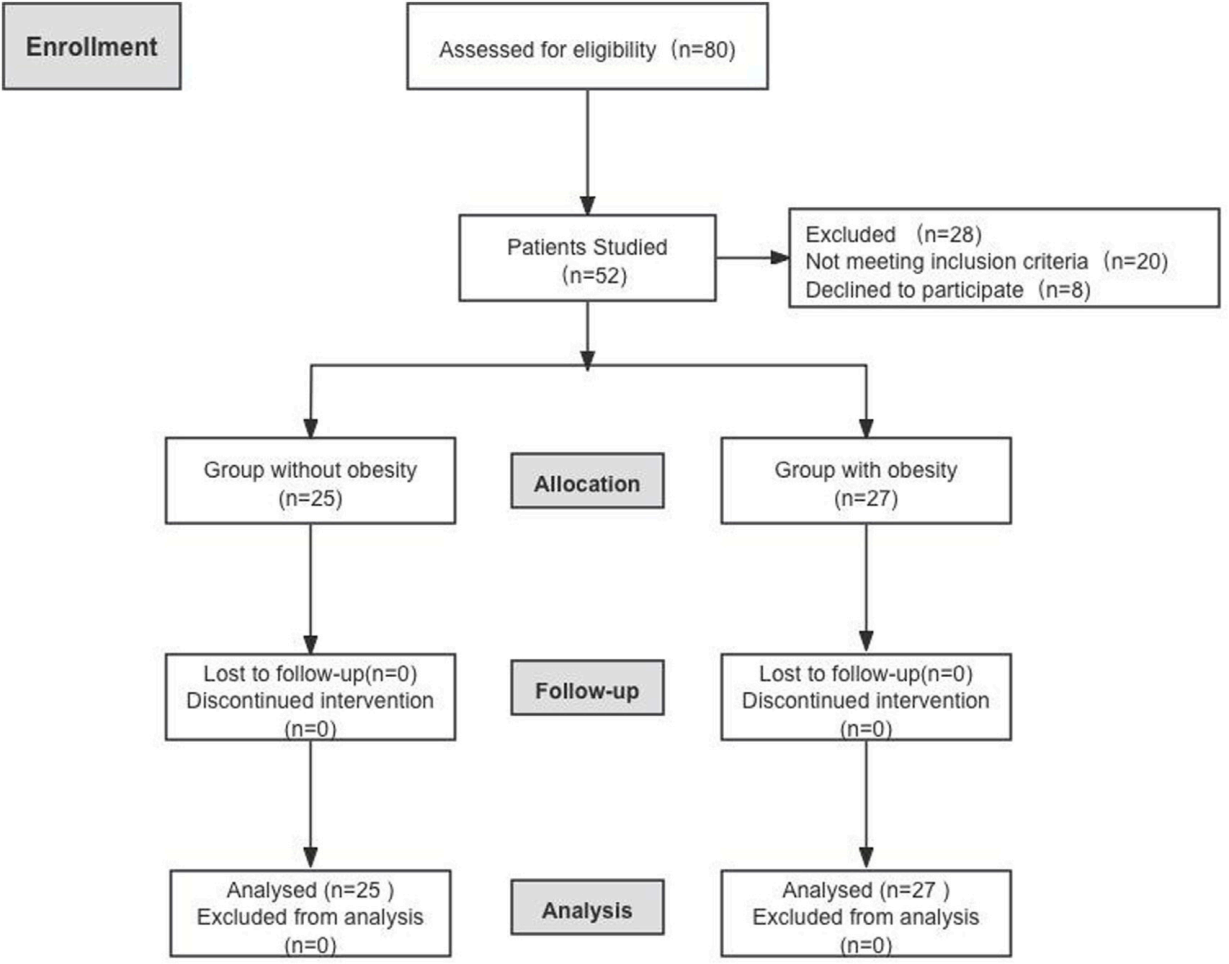
Consolidated Standards of Reporting Trials flow profile.

We enrolled 80 patients undergoing painless gastroscopy between August 2023 and December 2023, and 52 subjects (27 with obesity and 25 with normal weights) completed the study after obtaining informed written consent (Enrolment flow diagram; Figure 1).

The baseline characteristics table provides a comprehensive overview of the study population. The distribution of weight, BMI, and Mallampati airway grade among the groups revealed notable differences. Additionally, the sufentanil usage also differed significantly between the two groups and the duration of anesthesia induction was found to be significantly longer in the patients without obesity compared to those with obesity (Table 1).

**TABLE 1:** Participant characteristics.

Characteristic	Without obesity (n = 25)	With obesity (n = 27)	*p*-value
Sex, n (%)			0.098
Female	15 (60.0%)	10 (37.0%)	
Male	10 (40.0%)	17 (63.0%)	
Age, Mean ± SD, y	51 ± 13	48 ± 11	0.359
Height, Median (IQR),cm	165 (159, 172)	170 (159, 175)	0.595
Weight, Mean ± SD, kg	64 ± 11	91 ± 10	<0.001
BMI, Median (IQR), kg/m2	22.1 (21.5, 24.1)	32.1 (31.1, 33.8)	<0.001
Mallampati airway grade, n (%)			<0.001
1	7 (28.0%)	0 (0.0%)	
2	16 (64.0%)	8 (29.6%)	
3	2 (8.0%)	18 (66.7%)	
4	0 (0.0%)	1 (3.7%)	
Duration of anaesthesia induction^a^, Median (IQR), s	60 (40, 60)	60 (60, 90)	0.008
Duration of gastroscopy, Median (IQR), min	4.00 (4.00, 5.00)	5.00 (4.00, 5.00)	0.332
Recovery time^b^, Mean ± SD, min	8.08 ± 2.74	7.85 ± 2.30	0.747
Sufentanil, Median (IQR), ug	6.00 (6.00, 7.00)	9.00 (8.00, 10.00)	<0.001
Cipropol, Mean ± SD, mg	19 ± 6	22 ± 6	0.031

^a^ The interval from ciprofol administration to MOAA/S ≤ 1.

^b^ The interval from last ciprofol administration to MOAA/S score of 5.

**FIGURE 2:**
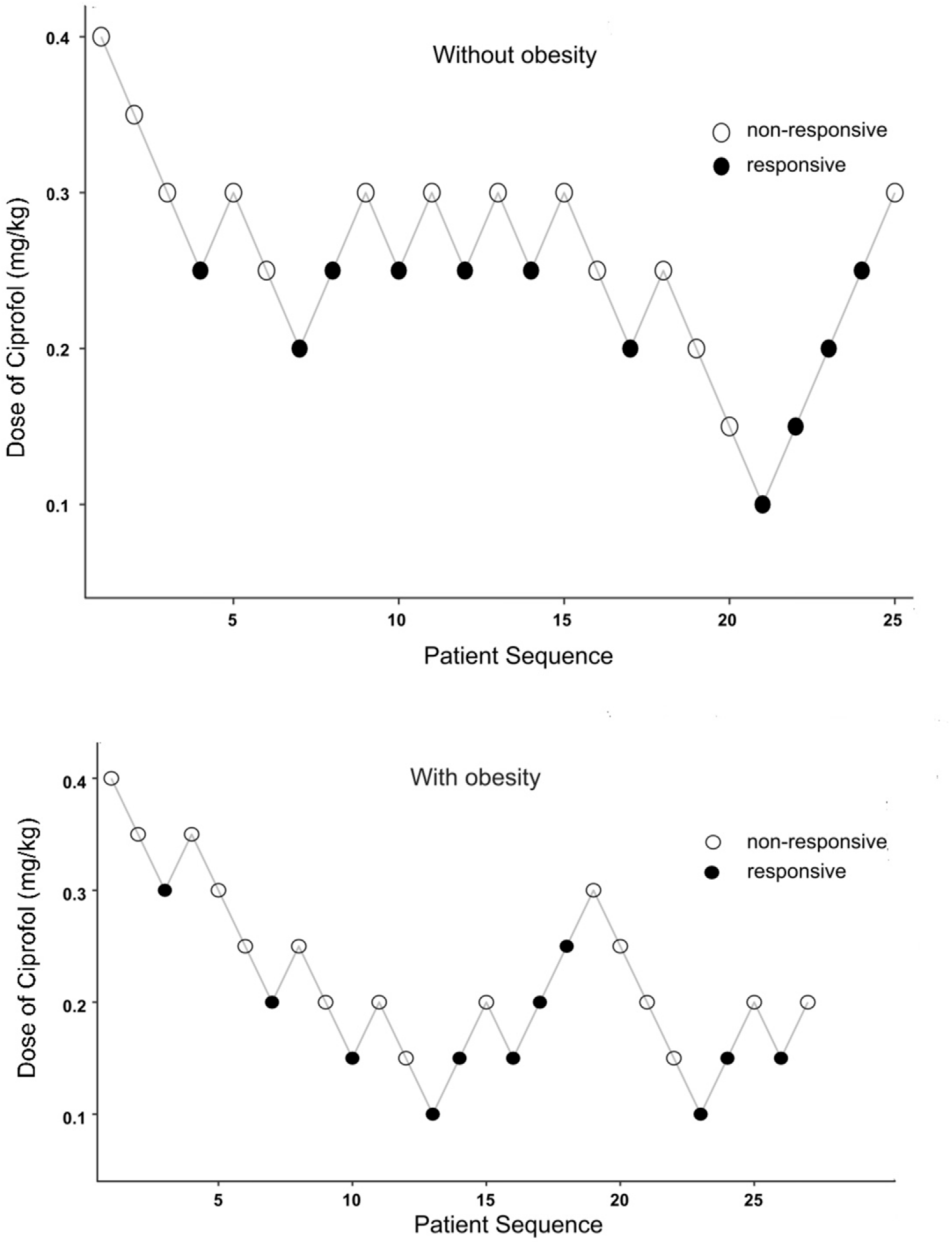
Dixon’s up-and-down sequential allocation response in patients with obese or nonobese after an initial coprofol bolus. The initial dose in both groups was 0.4 mg/kg, and the incremental change was 0.05 mf/kg. The seven midpoints of each group crossed from a “non-responsive” (empty circle) to “responsive” (filled circle) by coughing, choking, or any physical movement during the placement of the gastroscope.

**FIGURE 3:**
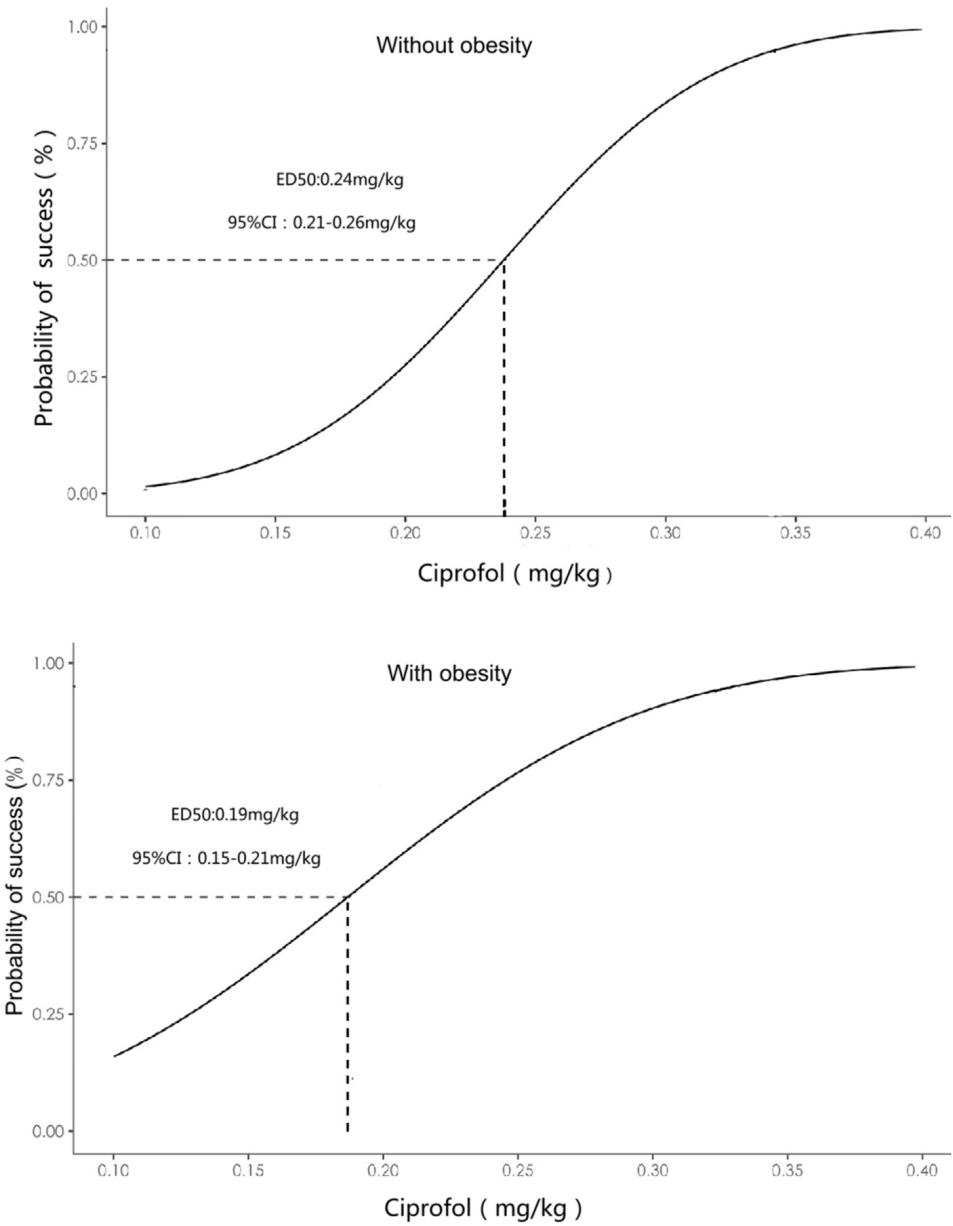
Dose-response curve of ciprofol combined with sufentanil inhibiting response to gastroscope insertion in patients with or without obesity plotted from the estimated probabilities of an effective response (1%–100%) calculated using probit analyses and bootstrapping methods.

The primary outcome ED50 of ciprofol combined with sufentanil inhibiting response to gastroscope insertion, using the Dixon up-down sequential method and calculated by probit analyses and bootstrapping methods, was 0.186 mg/kg with 95% CI of 0.153∼0.209 mg/kg in patients with obesity, and 0.237 mg/kg with 95% CI of 0.206∼0.253 mg/kg in patients without obesity (P < 0.05); (Figures 2, 3).

Table 2 shows a comparison of the occurrence of adverse events between the two groups, with hypoxemia occurring significantly more in the obese group than in the non-obese group.

**TABLE 2:** Adverse events of secondary outcomes.

Characteristic n (%)	Without obesity (n = 25)	With obesity (n = 27)	*p*-value
Hypoxemia	1 (4.0%)	9 (33.3%)	0.012
Hypotension	2 (8.0%)	1 (3.7%)	0.945
Bradycardia	1 (4.0%)	0	0.969
Choke	2 (8.0%)	4 (14.8%)	0.670
Apnoea	3 (12.0%)	1 (3.7%)	0.341
Satisfaction^a^			
Patient			0.241
0	0	0	
1	5 (20.0%)	2 (7.4%)	
2	20 (80.0%)	25 (92.6%)	
Endoscopist			0.317
0	2 (8.0%)	0 (0.0%)	
2	19 (76.0%)	24 (88.9%)	
1	4 (16.0%)	3 (11.1%)	
Anesthesiologist			0.189
0	3 (12.0%)	0 (0.0%)	
1	2 (8.0%)	3 (11.1%)	
2	20 (80.0%)	24 (88.9%)	

^a^ Satisfaction rating: 0: dissatisfied; 1: satisfied; 2: very satisfied.

**FIGURE 4:**
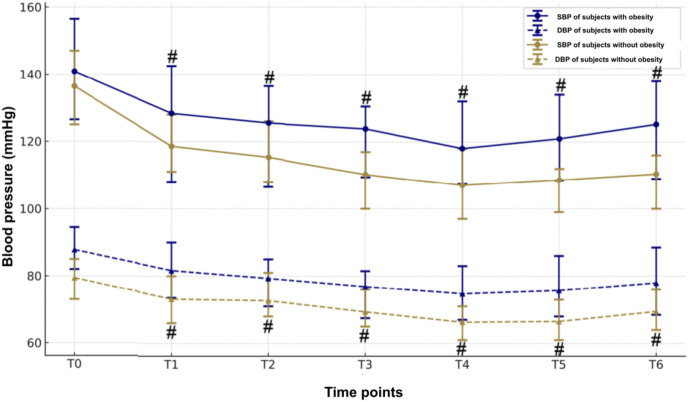
Blood pressure changes perioperatively between the two groups.

**FIGURE 5:**
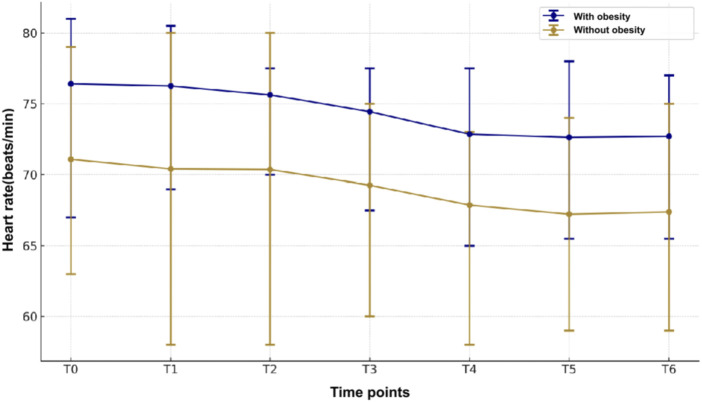
Heart rate changes perioperatively between the two groups.

**FIGURE 6:**
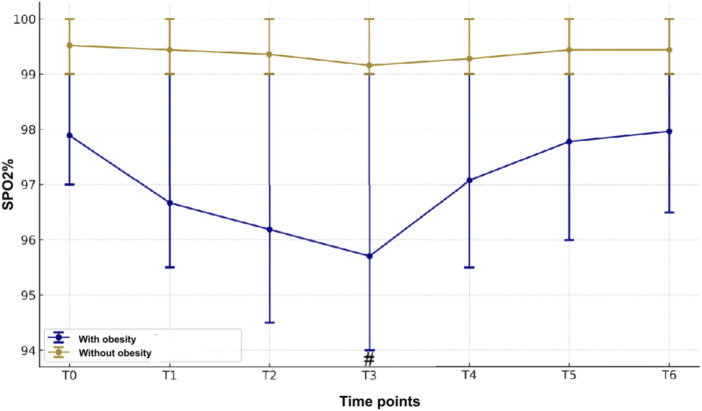
Evolutions of SpO2 perioperatively between two groups.

Patients with obesity had a markedly higher BP, including systolic and diastolic BP in comparison with the patients without obesity (Figure 4). Both groups had a marked fall in systolic and diastolic BPs from baseline to 1 min after ciprofol induction, which lasted up to the end of withdrawal of gastroscope. Notably, there is no significant decline in HR between the obese and normal weight groups at all time points (Figure 5), indicating that the BP was more sensitive to the effect of ciprofol. Oxygen saturation decreased significantly at 3 min after gastroscopy in obese patients compared with nonobese patients (Figure 6), which continued to improve until after the withdrawal of the gastroscopy.

## 4 Discussion

Anesthetics need to be titrated to the optimal dose for patients with different body weights. ED50 is a commonly used parameter for studying the dose-effect relationship of a drug and is typically considered the initial dose for clinical trials. Dixon’s up-and-down method is commonly used to calculate the dose-response relationship for determining the ED50 of a drug. The significant finding of the current study is that the ED50 of ciprofol combined with sufentanil inhibiting response to gastroscope insertion in patients with obesity was 0.186 mg/kg with 95% CI of 0.153∼0.209 mg/kg, and the patients with nonobese was 0.237 mg/kg with 95% CI of 0.206∼0.253 mg/kg. Thus, the 95%CIs barely overlapping, indicating that a significant difference in ED50 between the two groups. This finding also suggests that the dosage of ciprofol should be reduced for the obese population for its clinical application.

There are some reasons for the above findings. Firstly, alterations in the processes of absorption, distribution, metabolism, and excretion in obese individuals may contribute to the reduced required dose (Cortínez et al., 2010). Obese patients often experience changes in gastrointestinal function and blood flow, which can impact drug absorption, leading to variations in the drug’s bioavailability (Gomes et al., 2018). Additionally, obesity is typically associated with changes in cardiovascular function, including variations in cardiac output and hemodynamics, which may result in an increased blood volume. This increased blood volume can alter the distribution of the drug in the circulatory system, potentially affecting its effectiveness, meaning a lower dose of ciprofol may be needed to achieve the same anesthetic effect. Furthermore, obesity can lead to higher plasma levels of fatty acids, which compete with ciprofol for binding sites on plasma proteins. This competition results in increased concentrations of free (unbound) ciprofol, which is the pharmacologically active form of the drug, allowing lower doses to produce sufficient anesthesia. In addition, the larger amounts of adipose tissue in obese individuals can serve as a reservoir for lipophilic drugs like ciprofol, increasing the distribution of the drug in the body and leading to higher tissue concentrations, thus reducing the need for higher doses. Obesity may also influence hepatic enzyme activity, potentially decreasing the liver’s ability to clear ciprofol, thereby prolonging its presence in the body and reducing the required dose for effective anesthesia. Finally, obesity-related changes in drug metabolism and elimination pathways may further alter the sensitivity and response to the drug. Collectively, these factors contribute to the lower ED50 of ciprofol in obese patients.

It should be noted that for highly lipophilic drugs, the optimal volume descriptor may be total body weight or other metrics that include measures of adipose tissue. Adipose tissue is known to influence drug distribution and can significantly affect the volume of distribution for lipophilic drugs (Hinson et al., 2022). Another study demonstrated that lean body weight (LBW)-based induction dose of propofol did not provide adequate loss of consciousness in obese patients, and a majority of the patients in the LBW group required additional propofol to achieve adequate loss of consciousness (Subramani et al., 2017). Therefore, using total body weight descriptors may better reflect drug distribution and help optimize dosing strategies.

Currently, few studies have examined the safety and efficacy of sedation with ciprofol in obese patients. For sedation during gastrointestinal endoscopy, ciprofol-alfentanil reduced the e respiratory depression events compared with propofol-alfentani (Zhang J. et al., 2024). A recent meta-analysis demonstrated similar clinical efficacy and a better safety profile of ciprofol compared to propofol for sedation in gastrointestinal endoscopic procedures in adults. In addition, patient satisfaction scores were higher for propofol. (Ortegal et al., 2024). The effectiveness and safety of ciprofol have been confirmed, and it is now widely used in clinical practice. Clinical studies have explored the median effective dose of ciprofol in specific populations, including frail elderly individuals, pediatric patients, and immunocompromised patients (Yuan et al., 2024; Zhang X. et al., 2024; Pan et al., 2024). However, due to the relatively short time since ciprofol was introduced to the market, there is currently no established reference dose for its use in obese patients. This study identified a notable disparity in the dosage of sufentanil administered to the obese and non-obese groups, which can be attributed to the variations in body weight between the two groups. The obese group necessitated a higher dosage of sufentanil, potentially influencing the lower median effective dose (ED50) of ciprofol observed in these patients. The weight difference between obese and non-obese patients can impact drug distribution and metabolism, potentially influencing the drug response and dosage requirements. In this study, sufentanil was chosen as the pre-anesthetic medication, which is known to reduce anesthetic requirements and other complications when used in combination with low doses of opioids during intravenous anesthesia. Studies have indicated that obesity increases the volume of distribution and clearance rate of sufentanil but does not significantly affect its elimination rate compared to nonobese patients (Schwartz et al., 1991). This suggests that using a low dose of sufentanil in obese patients can be advantageous. Therefore, in this study, a combination of 0.1 μg/kg sufentanil and ciprofol was used as the sedation protocol for painless gastroscopy in obese patients.

In comparison to the non-obese group, obese patients exhibited a prolonged onset time of ciprofol. This observation can be attributed to several potential factors. Firstly, the increased presence of adipose tissue in obese individuals serves as a reservoir for lipophilic drugs like ciprofol, leading to delayed drug distribution and onset of action. (Dong et al., 2016). seconds, the altered blood flow and tissue perfusion commonly observed in obese patients may also contribute to the delayed effects of the drug (Cortínez et al., 2010; Gomes et al., 2018).

Both groups had a marked fall in systolic and diastolic BPs from baseline to 1 min after ciprofol, at the lowest level at 3 min, which lasted until the awakening observation period.

However, no significant fluctuations in heart rate were observed. This suggests that blood pressure is more sensitive to the effects of ciprofol than heart rate. One possible explanation for this difference is that ciprofol has a greater impact on peripheral vascular resistance, leading to a more pronounced reduction in blood pressure. Additionally, alterations in adipose tissue distribution and vascular function in obese patients may further contribute to increased sensitivity to the blood pressure-lowering effects of ciprofol.

Significant decreases in blood oxygen saturation were observed in the obese group following the administration of ciprofol. This finding raises important considerations for the safety and respiratory effects of ciprofol in obese patients undergoing painless gastroscopy. Several factors may contribute to the decreased blood oxygen saturation in obese patients, including the presence of obstructive sleep apnea (OSA) and ventilation-perfusion mismatch. Obese individuals are more prone to developing OSA, which can lead to episodes of hypoxia (Drager et al., 2013; Redline et al., 2023; Ji et al., 2022). Additionally, the altered distribution of adipose tissue in the upper airway can contribute to airway obstruction and further exacerbate respiratory impairments (Bonsignore, 2022; Murphy and Wong, 2013). To better understand and address these risks, we plan to conduct a randomized controlled study specifically investigating respiratory depression associated with the use of ciprofol during painless gastroscopy in obese patients.

There are some limitations in this study. Firstly, this study only explored the ED50 of ciprofol for the loss of consciousness inhibiting response to gastroscope insertion in patients with obesity and nonobesity patients and did not conduct a randomized controlled trial based on age, sex and comorbid conditions stratification to explore these variables and provide more comprehensive recommendations for dose adjustments based on these individual differences. Secondly, the study only included obese patients with a BMI range of 30–40, excluding morbidly obese patients (BMI >40). This was done due to the relative contraindication of intravenous anesthesia in morbidly obese patients (Hinson et al., 2022). However, it is important to acknowledge that the anesthesia management and requirements for morbidly obese patients may differ significantly. Future studies are needed to explore the dosage requirements of ciprofol for morbidly obese patients in painless gastroscopy. Thirdly, we did not investigate the use of ciprofol as a standalone agent in either obese or non-obese patients during painless gastroscopy. The choice to combine ciprofol with opioids, such as sufentanil, was made to reduce the overall anesthetic requirement and enhance anesthesia safety, particularly in obese patients.

## 5 Conclusion

In conclusion, our findings demonstrate the ED50 values of ciprofol combined with low-dose sufentanil for inhibiting response to gastroscope insertion in patients with obesity were lower than in patients without obesity. These results underscore the importance of tailored anesthetic dosing strategies based on patient weight categories to optimize procedural sedation efficacy and safety.

## Data Availability

The raw data supporting the conclusions of this article will be made available by the authors, without undue reservation.
